# Clinical Research Progress of the Post-Stroke Upper Limb Motor Function Improvement via Transcutaneous Auricular Vagus Nerve Stimulation

**DOI:** 10.1155/2023/9532713

**Published:** 2023-09-25

**Authors:** Xiaolong Shi, Jingjun Zhao, Shutian Xu, Meng Ren, Yuwei Wu, Xixi Chen, Zhiqing Zhou, Songmei Chen, Yu Huang, Yuanli Li, Chunlei Shan

**Affiliations:** ^1^Department of Rehabilitation Medicine, Tong Ren Hospital, Shanghai Jiao Tong University School of Medicine, 200336, Shanghai, China; ^2^Institute of Rehabilitation, Shanghai Jiao Tong University School of Medicine, 200025, Shanghai, China; ^3^School of Rehabilitation Science, Shanghai University of Traditional Chinese Medicine, 201203, Shanghai, China; ^4^Engineering Research Center of Traditional Chinese Medicine Intelligent Rehabilitation, Ministry of Education, 201203, Shanghai, China; ^5^Department of Rehabilitation Medicine, Yueyang Hospital of Integrated Traditional Chinese and Western Medicine, Shanghai University of Traditional Chinese Medicine, 200437, Shanghai, China; ^6^Shanghai No.3 Rehabilitation Hospital, 200436, Shanghai, China

## Abstract

Stroke is a disease with high morbidity and disability, and motor impairment is a common sequela of stroke. Transcutaneous auricular vagus nerve stimulation (taVNS) is a type of non-invasive stimulation, which can effectively improve post-stroke motor dysfunction. This review discusses stimulation parameters, intervention timing, and the development of innovative devices for taVNS. We further summarize the application of taVNS in improving post-stroke upper limb motor function to further promote the clinical research and application of taVNS in the rehabilitation of post-stroke upper limb motor dysfunction.

## 1. Introduction

Stroke is a highly morbid and disabling disease that places a significant burden on healthcare globally [1, 2]. As the second leading cause of death in the world, the estimated global cost of stroke has exceeded $721 billion [3], and the majority of post-stroke survivors experience sequelae of upper limb motor impairment after the onset of stroke [4, 5]. The treatment of stroke requires the application of multiple therapeutic strategies [6]. And in recent years, with the development of various types of technology, the development of electronic devices to modulate abnormal neural activity by the directional delivery of electrical stimulation has become increasingly sophisticated [7]. Neuromodulation modalities have begun to play a more important role in post-stroke rehabilitation. Currently, innovative rehabilitation treatments such as robotics, brain computer interfaces, wearable technology, and virtual reality are applied to stroke survivors [4, 8, 9]. Notwithstanding rehabilitation treatments, a considerable population of stroke survivors worldwide continue to endure long-term disabilities.

Vagus nerve stimulation (VNS) is an invasive stimulation technique that requires the surgical placement of a stimulation device inside the patient's body and was first approved by the US Food and Drug Administration (FDA) as a treatment for intractable epilepsy [10]. In recent years, there has been a growing trend to apply it to functional recovery in stroke rehabilitation [11, 12]. VNS combined with rehabilitation showed better motor function outcomes in stroke patients in comparison to mental health outcomes [13], and many clinical trials showed that VNS can improve upper limb motor function in patients with post-stroke upper limb motor dysfunction [14–16]. Since Ventureyra [17] first introduced the concept of vagus nerve stimulation through the skin of the ear in 2000, transcutaneous auricular vagus nerve stimulation (taVNS) is thought to be a potentially more efficient and safer alternative noninvasive therapy than VNS based on several post-stroke upper limb impairment studies in recent years [18–20]. taVNS provides electrical stimulation to the auricular branch of the vagus nerve (ABVN) by placing electrode pads in the cymba conchae. Modern neuroanatomical findings have shown that the visceral auricular region of the human ear is the only area of the body surface where the vagus nerve is distributed [21]. The ABVN has fibers that project directly to the nucleus of the solitary bundle of the sensory relay nucleus and make synaptic connections with brain regions, such as the locus coeruleus, parabrachial nucleus, hypothalamus, amygdala, and hippocampus. Based on this, researchers have combined modern neuromodulation techniques and proposed an innovative approach to replace implantable vagus nerve stimulation with taVNS. Given that invasive VNS requires surgical device implantation into the body, many stroke patients with co-morbidities and chronic disease may be unwilling or unsuitable for it as a first choice for rehabilitation. Therefore, it is necessary to determine whether taVNS combined with rehabilitation can also promote neuroplasticity and improve clinical outcomes in stroke patients. This paper provides a brief review of the clinical application of taVNS for post-stroke motor function rehabilitation.

## 2. Stimulation Parameters

As basic science and clinical trials related to taVNS have been conducted, the issue of studying its optimal stimulation parameters becomes increasingly important [22]. The optimal stimulation parameters for taVNS in stroke patients remain unknown, and the observed efficacy of taVNS is not as significant as VNS when using the parameter settings from previous VNS studies [23, 24]. Therefore, subsequent studies should explore the parameter settings applicable to taVNS rather than simply following the optimal stimulation protocol used in VNS studies. There is no specific study on the stimulation parameters of taVNS, while the parametric studies performed for VNS mainly include current intensity, pulse width, intervention duration, and stimulation interval.

Only one study used a fixed current intensity of 0.5 mA [25], while other related studies of taVNS used more individualized approaches for current intensity settings (see Table 1). The current tendency in clinical trials is to adjust the stimulation intensity of taVNS to the highest level that the subject can tolerate to achieve the best therapeutic effect [18–20, 26], or use a current intensity slightly below the patient's pain threshold [27–29]. The difference between the two descriptions is that the former expression implies that a certain amount of pain may be tolerated within the subjects' tolerance, whereas the latter one implies that the subjects do not feel pain at all. No current studies have used both methods, and the results of the respective studies suggest that both fixed and individualized current intensity settings contribute to the rehabilitation of upper limb movement impairment after stroke. When available, it is recommended that the parameters be personalized according to the characteristics of each subject.

As to the setting of pulse width, the main choices in stroke clinical motor disorder treatment are 0.1 ms [19, 20, 27] and 0.3 ms [18, 26, 28, 29]. Stimulation frequency includes 20 Hz [18, 25, 26, 29], 25 Hz [19, 20, 27], and 30 Hz [28]. Despite the varying stimulation parameters, all of the mentioned taVNS studies showed a significant improvement in the participants' upper limb function. Future research can build upon the above-mentioned VNS studies to explore the optimal stimulation parameters for taVNS intervention in post-stroke motor dysfunction.

## 3. Timing of taVNS and Rehabilitation Training for Post-Stroke Intervention

Recovery of motor function in clinical stroke patients is often accompanied by rehabilitation. Therefore, it is crucial to determine the optimal timing of taVNS treatment in conjunction with rehabilitation. At present, there are no relevant reports regarding taVNS intervention timing in clinical rehabilitation protocols. Given the potential of taVNS to improve motor function in stroke patients, it is important to identify the most effective timing and sequencing of taVNS treatment.

Although there are no studies regarding the optimal timing of intervention when taVNS is applied to post-stroke patients, we can still summarize the current trends in intervention timing from the available clinical studies. Two current clinical studies regarding taVNS intervention for post-stroke upper limb motor dysfunction did not highlight a specific temporal relationship between taVNS and conventional rehabilitation [25, 27]. Rehabilitation training is often performed immediately after taVNS treatment [18, 26, 29]. Li et al. [26] randomized 60 acute stroke patients to a taVNS group and a control group and gave them a 4-week course of 20-minute treatment five times per week (20 times in total). The patients received rehabilitation therapy as soon as they finished the real or sham taVNS session. In another study, 14 patients with chronic stroke also received robotic training immediately after the end of active or control taVNS [29]. Three other studies clearly indicated that rehabilitation training and taVNS were synchronized, including both conventional and rehabilitation robotic training [19, 20, 28]. For example, Baig et al. [20] performed functional arm exercises 300 times on average in 12 patients with ischemic stroke while simultaneously treating these patients with maximal tolerated intensity of taVNS. Chang et al. [28] administered upper extremity robotic training to 34 chronic stroke patients with upper extremity hemiparesis and gave active taVNS or sham taVNS at the onset of visual cues for stretching movements. As to VNS clinical studies on post-stroke upper limb motor dysfunction, the mainstream method is to offer patients VNS at the same time of rehabilitation treatments [14, 15, 22, 30]. The temporal sequence of stimulation is important and may have a significant impact on the rehabilitation outcome of clinical patients. Although all of the mentioned taVNS studies showed significant improvement of upper limb motor function after treatment, no comparison of the clinical effects of different intervention timings was conducted in a single taVNS study. Therefore, further studies are needed to investigate the optimal timing of taVNS stimulation in relation to rehabilitation training.

## 4. Progress in the Development of taVNS Devices

A previous review classified the types of taVNS devices [31], which can be simply divided into open-loop and closed-loop taVNS devices. Closed-loop taVNS is defined as a closed-loop system modulated by biofeedback signals to achieve automatic control of taVNS treatment. It primarily consists of a bio-signal sensor and a taVNS stimulator that can rapidly adapt to changing conditions to provide individualized taVNS treatment for every patient to achieve increased treatment efficiency, improved quality of life, and reduced severity of side effects [32].

Current clinical studies of taVNS for improving post-stroke upper limb motor function mostly use open-loop devices [18–20, 25–29]. These commercially available taVNS devices do not consider patient's real-time physiological status and, thus, are not individualized. As such, it is difficult to achieve optimal treatment results. As previously noted, a universally accepted optimal stimulation protocol for taVNS in the management of post-stroke motor dysfunction is presently absent. Even if forthcoming research identifies the most effective stimulation parameters for this cohort, these parameters may not be applicable to an individual patient's particular condition at various junctures. In view of this exigency, closed-loop systems are deemed a propitious therapeutic modality. The closed-loop taVNS system avoids over-stimulation and under-stimulation of the patients and improves treatment by adjusting the stimulation parameters at any time according to individualization. The feasibility of a closed-loop design of neuromodulation devices has been demonstrated [33–37], and the concept of closed-loop taVNS is gradually being formally applied in scientific research [38–40].

In this closed-loop system, different biofeedback signals should be selected based on the disease being treated. These biofeedback signals must meet the conditions necessary to trigger the closed-loop system and do not interfere with other functions. At present, the most suitable closed-loop taVNS system for motor function rehabilitation in stroke patients is motor-activated auricular vagus nerve stimulation (MAAVNS), which uses electromyography (EMG) to detect muscle movements to trigger taVNS stimulation [39, 40], thereby maximally avoiding errors that may be caused by artificially initiated stimulation.

This technique has been successfully applied to improve neonatal oral motor coordination [40]. It can be extended to any motor rehabilitation paired with taVNS. The technique requires that target muscle movements be isolated and individual muscle activation be quantified via EMG. Furthermore, the electroencephalogram (EEG) gated closed-loop taVNS system has great potential [41, 42]. In particular, when patients present with severe hemiparesis, EEG can identify the patient's intention to move the affected limb, which may compensate for the limitations of EMG in capturing the full range of muscle activity. More recently, a closed-loop taVNS device that can be synchronized with respiration and heartbeat has been developed [43] and can achieve an accuracy of ±100 ms. One of the main benefits of this closed-loop device is that the stimulation parameters can be adjusted by monitoring the subject's physiological signals in real-time, thus improving the comfort and feasibility of the subject receiving taVNS treatment during the exercise training.

In addition, a device under development attempts to use vibrotactile stimulation instead of electrical stimulation [44], also through the cymba conchae, to modulate the vagus nerve. While this device has not yet been studied for efficacy in any disease, it gives us a new idea for VNS device design. If taVNS is used in the future in conjunction with a brain computer interface (BCI), vibrotactile stimulation could avoid the EEG noise generated by electrical stimulation, allowing for more accurate measurement of the signal for BCI applications. Future development in taVNS devices can be summarized as (1) coupling with other assessment devices and adding biofeedback to improve stimulation parameters in real time, and (2) improving the stimulation modalities (or materials in contact with body parts) to increase patient comfort during use.

## 5. Clinical Effect of taVNS on Post-Stroke Upper Limb Motor Dysfunction

In terms of assessing the clinical efficacy of taVNS interventions for post-stroke motor dysfunction, most of the relevant clinical studies have used several evaluation indicators (see Table 1). Among these, the upper limb Fugl-Meyer Assessment Scale (FMA-U) is the most commonly used and is considered to be the core outcome measure of stroke recovery [45]. It is more convincing in reflecting the recovery outcome of stroke patients [46]. Most studies have considered that an increase in FMA-U scores after treatment reflects the improvement of post-stroke upper limb motor function due to taVNS treatment [18–20, 25–29]. Current studies have shown that rehabilitation therapy combined with taVNS is significantly more effective than the control group [18, 25–27]. Since the sinus node is mainly innervated by the right vagal nerve fibers [47], most studies have chosen to stimulate only the left ear to reduce the risk of causing cardiac side effects.

In a prospective study, 13 patients suffering from ischemic stroke for at least 3 months with residual upper limb dysfunction were subjected to a 6-week repetitive upper limb training task combined with a taVNS intervention [19]. The parameters of the taVNS intervention were set as follows: stimulation frequency of 25 Hz, pulse width of 0.1ms, and current intensity using the maximum value that the patient could tolerate. The FMA-U scale, Action Recovery Arm Test (ARAT), Modified Rankin Score (MRS), Barthel Index (BI), Stroke Impact Scale (SIS), and EEG were used for assessment. The results showed that taVNS is feasible and well tolerated in post-stroke rehabilitation in combination with repetitive upper limb training. In a subsequent study [20], this team used the same intervention mentioned in the previous study and found that the combination of taVNS and motor rehabilitation could improve sensory recovery in chronic stroke patients. The overall rehabilitation effect of simultaneous sensory and motor recovery may be better. taVNS in these studies used a novel method [19, 20], with the physical therapist holding the on/off button, stimulating when the patient starts to move their arm and stopping the stimulation when the movement stops. This type of stimulation is similar to closed-loop stimulation [32], but it is more prone to human error in comparison. Therefore, it can be used as a transitional means, but closed-loop systems are expected to be gradually adopted in the future when the technology is mature.

At present, a total of two studies have used rehabilitation robotic training instead of conventional rehabilitation combined with taVNS to intervene in post-stroke motor dysfunction [28, 29], one of which recruited 34 chronic stroke patients and randomized them to a trial group and a sham stimulation group for 3 weeks treatment [28]. The trial group was trained with the upper extremity rehabilitation robot while performing taVNS stimulation, and taVNS stimulation was performed automatically whenever movement of the upper extremity extension started. The stimulation is initiated similar to a closed-loop system, as soon as the patient is able to complete the movement actively, based on light cues, while for more severe patients, the robot assists to passively complete the training. The current intensity was slightly below the patient's pain threshold (0.1–5 mA) at 30 Hz with a pulse width of 0.3 ms [28]. The sham stimulation group evaluated the current intensity threshold only before each treatment, and the stimulation intensity was reduced to 0 during robot training. Surface EMG, Wolf Motor Function Test (WMFT), and Modified Tardieu Scale (MTS) were used as efficacy evaluation indicators. The results showed an improvement in behavioral tests in both groups after 3 weeks of treatment, while patients in the trial group showed a more significant reduction in hand and wrist spasticity and a significant increase in peak surface EMG amplitude during extension. The EMG results indicated that taVNS could mitigate spasticity and increase motor control. This suggests that the peak surface EMG amplitude of the biceps may be a potential biofeedback signal for taVNS treatment.

There are several kinds of side effects, including skin redness, nausea, vomiting slight pain and light-headedness. In the two studies of taVNS combined with conventional rehabilitation training, three participants had skin redness [18, 26]. In another study of taVNS combined with repetitive task practice, one participant experienced light-headedness [19]. Additionally, in a study of taVNS combined with conventional rehabilitation training, five participants reported experiencing nausea, vomiting, or slight pain in the ear [27]. Conversely, two studies of taVNS combined with robotic training and another study of taVNS combined with conventional rehabilitation training reported no side effects [25, 28, 29]. All of the studies that measured parameters of heart rate and blood pressure demonstrated that there were no significant effects caused by taVNS on these parameters [19, 26, 29].

Current studies have difficulty in harmonizing the accompanying treatments used in different researches. In fact, it is understandable that for each patient the therapist should determine the appropriate individual treatment protocol. For the researchers this also makes it more challenging to control for variables. However, the available studies show that the effect of taVNS on stroke is significant regardless of treatment timing and accompanying treatments [18, 19, 25, 26, 28, 29].

## 6. The Stimulation Site of taVNS

In current clinical studies (as shown in Table 1), the cymba conchae has been selected for stimulation and is widely considered by researchers to be the optimal site for taVNS stimulation [48, 49]. However, a recent study reported no significant between-group differences in indicators when the tragus, cymba conchae, and earlobe were used as different targets for taVNS, which led the researchers to question the validity of using the earlobe as a sham-stimulation target [50]. Although some studies still use the earlobe for sham-stimulation [29], the most common sham-stimulation method used in existing studies is to place electrodes at the treatment site and without performing electrical stimulation. While only 45% of the tragus is innervated by the ABVN, 100% of the cymba conchae is innervated by the ABVN [21]. Despite the experimental results mentioned not supporting the expectation that the cymba conchae is the superior treatment site compared to the tragus, we should continue exploring the differences between these two sites as potential treatment targets in clinical motor function rehabilitation for stroke patients.

## 7. Conclusions and Outlook

Clinical research work on taVNS to improve motor function in stroke patients is still in its infancy. Most of the existing studies are limited by small sample size, inconsistent stimulation parameters, and lack of follow-up or short follow-up time. Therefore, randomized controlled clinical trials on the optimal timing and stimulation parameters of taVNS application in stroke patients should be conducted in the future with reference to the patterns found in existing basic VNS studies to determine the treatment protocols with the optimal therapeutic efficacy. A previous review systematically summarized the parameter settings of taVNS applied to different diseases [31], but there are no clinical studies on the timing and stimulation parameters of taVNS applied to the treatment of post-stroke motor dysfunction, and no relevant basic research has been conducted to study the timing and stimulation parameters of taVNS.

In terms of safety, the side effects of taVNS have been greatly reduced as compared with VNS, but a few adverse events that may be associated with taVNS (e.g., mild nausea, vomiting, and mild ear pain [27]) still occur, and subsequent studies could focus on this to address concerns for clinical use.

Currently available clinical studies of taVNS interventions for post-stroke motor function are focused on upper extremity motor function, and no clinical studies related to lower extremity motor function have been conducted. The subject selection was mainly in ischemic stroke patients, with only three studies recruiting both hemorrhagic and ischemic stroke patients [26, 28, 29], and these three studies did not explore the difference in efficacy of taVNS in patients with ischemic versus hemorrhagic stroke. Based on the improvement of upper extremity motor function in stroke patients with taVNS combined with various rehabilitation therapies, future clinical trials could try to investigate the efficacy of taVNS interventions for lower extremity motor function rehabilitation, in addition to exploring the differences between ischemic and hemorrhagic stroke patients according to the different etiologies that trigger stroke and discovering the most applicable treatment paradigm.

Overall, taVNS is a very promising technology that has a wide range of applications in a variety of diseases. As a new technique, the therapeutic efficacy of multiple rehabilitation methods in combination with taVNS for post-stroke movement disorders has been initially confirmed. It is a future research direction to increase the follow-up time and explore the best treatment pattern for patients with different etiology and disease courses.

## Figures and Tables

**Table 1:** Clinical study of taVNS intervention for motor function after stroke.

Author (reference number)	Sample size (*N*)	Group	Stimulation site	Stimulation parameters and time	Course of treatment and follow-up	Assessment tools	Effects of taVNS	Type of stroke and motor impairment	Accompanying treatments
Wu et al. [18]	21	taVNS group and sham taVNS group	The cymba conchae of the left ear	600 pulses (20 Hz, each pulse duration 0.3ms), lasting 30 s each, stimulated every 5 min for 30 min per day. The intensity was selected according to the subject's individual body tolerance.	15 days, 12 weeks	FMA-U, WMFT, FIM, Brunnstrom	taNVS improves upper limb motor function in subacute ischemia stroke patients without obvious adverse effects.	Ischemia stroke; between 0.5 and 3 months postonset; single upper limb motor function impairment	Postural control, proprioception exercises, neuromuscular facilitation, and gait training
Zhu Lin et al. [27]	113	Control group and observation group	The cymba conchae of the left ear	The current intensity was the maximum value for which the patient did not feel pain. The stimulation interval was 30 s, rest 30 s, and the pulses were set in biphasic waves with 25 Hz and a wave width of 0.1ms for 20–30 min each time, 5 times per week.	1 month, none	FMA-U, MFAS, MBI	Occupational therapy combined with taVNS regulates the level of norepinephrine, acetylcholine, and dopamine, improve upper limb motor function.	Ischemia stroke; between 0.5 and 3 months postonset; single upper limb motor function impairment	Upper limb motor function and activity participation ability training
Redgrave et al. [19]	13	None	The concha of the left ear	The intensity was at the patient's maximum tolerance level, with a wave width of 0.1ms and 25 Hz. The stimulation was turned on when the patient began to move their arm, and the movements were repeated more than 300 times per treatment, three times per week.	6 weeks, none	FMA-U, ARAT, MRS, BI, SIS, Motor Activity Log, PHQ9, GAD7, Fatigue Assessment Scale	taVNS combined with upper limb repetitive movements is feasible, and improves upper limb function.	Ischemia stroke; at least 3 months postonset; upper limb motor function impairment	Large- range arm movements and repetitive task-specific movements training
Fioravante Capone et al. [29]	14	Randomly divided into sham stimulation group and stimulation group	The left external acoustic meatus at the inner side of the tragus	The pulse frequency was 20 Hz and the pulse duration was 0.3ms, which was repeated every 5 min for 60 min of continuous operation. A current intensity slightly below the patient's pain threshold.	10 days, 2 weeks	FMA, NIHSS, Rankin Scale, BI, Modified Ashworth Scale	taVNS combined with robotic training is feasible in stroke patients, and slightly improves upper limb function.	Ischemic or haemorrhagic stroke; at least 1 year postonset; hand function impairment	Robotic training delivered at proximal or distal segment of the affected limb according to the degree of impairment
Zhang Liping et al. [25]	42	Randomly divided into sham stimulation group and stimulation group	The cymba conchae of the hemiplegic side of the body	20 Hz square wave with a current intensity of 0.5mA. Each session lasted 30 s and was stimulated every 2 min. Each treatment lasted 30 min, once a day, five times a week.	3 weeks, none	FMA-U, WMFT, FIM	taVNS improves upper limb function with no obvious adverse effects.	Ischemia stroke; within 3 months postonset; hemiplegia	Internal medicine treatment and comprehensive rehabilitation training
Li et al. [26]	60	Randomly divided into taVNS group and control group	The left auricular cavum conchae	5 times a week, once for 20 min. 0.3ms square wave at 20 Hz for 30 s, repeated every 5 min. The current intensity (1.71 ± 0.5 mA) was adjusted according to the tolerance of each patient.	4 weeks, 1 year (as well as 1, 3, and 6 months after the start of treatment)	WMFT, FMA-U, FMA-L, FMA-S, SIS, HADS	taVNS combined with conventional rehabilitation training is safe and effective.	Ischemic or haemorrhagic stroke; within 1 month postonset	Postural control, neuromuscular facilitation and sensory integration exercises
Chang et al. [28]	34	Sham stimulation group and taVNS group	The left cymba conchae	30 Hz, with a pulse width of 0.3ms and a current intensity slightly below the patient's pain threshold (0.1–5 mA), 3 times a week for 1 hr each treatment.	3 days, 3 weeks	FMA-U, MRC, WMFT, MTS	taVNS combined with robotic training improves upper limb function.	Ischemic or haemorrhagic stroke; at least 6 months postonset; upper limb hemiparesis	Robotic training
BAIG et al. [20]	12	None	The concha of the left ear	Pulse width of 0.1ms, 25 Hz, and pulse amplitude as maximally tolerated by the participant.	6 weeks, none	FMA-U	taVNS combined with motor rehabilitation may improve sensory recovery.	Ischemia stroke; at least 3 months postonset; upper limb motor function impairment	Repetitive upper limb task training

*Notes:* ARAT: Action Recovery Arm Test, BI: Barthel Index, FIM: Functional Independence Measurement, FMA-L: Fugl–Meyer Assessment-Lower Limb, FMA-S: Fugl–Meyer Assessment-Sensory, FMA-U: Fugl–Meyer Assessment-Upper Limb, GAD7: Generalized Anxiety Disorder 7, HADS: Hospital Anxiety and Depression Scale, MBI: Modified Barthel Index, MFAS: Motor Function Assessment Scale, MRC: Medical Research Council Motor Power Scale, MRS: Modified Rankin Scale, MTS: Modified Tardieu Scale, NIHSS: National Institute of Health Stroke Scale, PHQ9: Patient-Health Questionnaire, SIS: Stroke Impact Scale, taVNS: Transcutaneous Auricular Vagus Nerve Stimulation, WMFT: Wolf Motor Function Test.

## Data Availability

No data were declared for this study.
